# Fifth, Sixth, Seventh and Eighth Districts Dental Societies of New York State

**Published:** 1889-02

**Authors:** 


ARTICLE VIII.

### FIFTH, SIXTH, SEVENTH AND EIGHTH DISTRICTS DENTAL SOCIETIES OF NEW YORK STATE.

One of the most pleasant and profitable conventions which has been held for a long time, was that of the union meetings in Syracuse, October 25th, 26th. The organizations under the skilful and genial leadership of Drs. S. B. Palmer and G. L. Curtis, was all that could be desired. Among the foreign visiting guests the following were present from Canada:—Drs. J. B. Willmott, G. S. Cæsar, C. V. Snellgrove, J. G. Roberts, and W. G. Beers. The paper of Dr. J. C. Curtis on "Chemistry an Important Feature in Dental Education," assumed a practical character. He maintained that all dental caries is due to external causes, the substances brought in contact with them as food or medicine, or those resulting from decomposition of one or both. If sour apple or lemon or dilute acid is taken into the mouth, the teeth become what is termed "set on edge," which means that the calcium of the tooth has been acted upon by the acid, forming a new compound, and leaving the organic parts unprotected. From decomposition of starchy foods results acetic acid, and from this by further fermentation, butyric acids. Nitrogenous substances, as lean meats, albumen and mucus, are converted by decomposition into acids of the nitric and nitrous groups. The normal period of the stomach contains hydrochloric acid, and in manv cases of dyspepsia butyric acid is found; while nitric, as sulphuric, sulphurous, nitro-hydrochloric, and hydrochloric acids are in use medicines. Of the organic acids, we have in apples, malic acid; in plums, prunes, grapes and currants, tartaric acid; in lemons, citric acid.

Whenever lean meats are allowed habitually to remain between teeth, decay ensues. This is explained by authorities as follows: "When nitrogenous organic matter is exposed to the air, the nitrogen assumes the form of ammonia; but when alkalies, such as potash, soda or lime are present, a further slow oxidation takes place, and nitrates of these metals are formed." In combatting the action of acids upon tooth-structure, an acid is always unsatisfied until it is combined with a base to produce a salt, so that instead of permitting the salt to be formed at the expense of tooth-structure, the base is supplied from outside, the problem is solved.

Happily, potassium, and magnesium have more affinity for acids than calcium. It is well established that an approximately decayed tooth, though ever so well filled, will again decay if subjected to the same influences as before it was treated. The conditions are changed in a measure by self-cleaning spaces, by contriving only the removal of one or more teeth; but whatever means are employed, reliance is still placed on the saliva, because, first it acts mechanically in conjunction with the tongue, and second it is alkaline and will neutralize acids with which it comes in contact. The teeth of smokers who take care of them are better preserved than those of non-smokers, because the salivary glands are stimulated to produce an abnormal quantity of saliva, and because a certain amount of creasote comes into the mouth with the smoke to retard fermentative action. Decay cannot occur in the presence of an alkali. Alkaline earths, as mouth-washes, are valuable.

Dr. Barrett admits the correctness of the merely chemical ideas, but they ignore later investigation. In the mouth we find the acids so diluted that the reactions do not always occur. Citric acid may produce the reaction noted, malic acid found in an apple is not strong enough to act on enamel. We used to talk of catalysis, but that question has been solved by the knowledge we now have of the fermentative organisms. Dr. Miller has shown that they produce acids, and that these are the cause of caries. Fermentation lies at the root of all dental chemistry.

Dr. Dwinelle said fermentation will account for much of the phenomena of caries which chemistry will not account for. He denied that decay is as apt to recur, as a rule, after the teeth are put into good condition.

Dr. Brophy disagreed with the statement that caries does not occur in alkaline conditions of the saliva. Some of most extreme cases of decay are found in mouths that are always alkaline. This is accounted for by the fact that lactic acid produced through the agency of fermentation is the most important factor in decay.

Dr. J. B. Willmott took exception to the explanation of the phenomenon of "setting the teeth on edge." He thought is a hyperæsthetic condition caused by the acids permeating the enamel, and acting as an irritant to the underlying dentine, the condition passing away as soon as the acids are washed out. He thought that it is only those acids which are formed in the mouth in actual contact with the teeth that act upon them, and that they act only when in the nascent condition; and that those acids which are taken into the mouth in fruits, etc., have no destructive action upon the tissues with which they are brought into contact. It has been pretty well established that the exciting cause of caries in the teeth is fermentative action, and that recurrence of caries after filling, may be better prevented by the use of germicides and antiseptic dentifrices than by the use of antacids.

Dr. J. C. Curtis believes where decay is found in alkaline mouths, there is a line which corresponds exactly with the location of the decay, where tests with litmus show that there are acids. He believes in the use of antiseptics —in those things which prevent fermentation—because they stop the formation of acids.

Dr. E. T. Darby referred to the experiments conducted by the late Dr. Wescott, showing the action of acids upon tooth structure. He found that all mineral and vegetable acids need not be of any great strength to decalcify teeth. Even one part in one hundred was sufficient in most cases to corrode the surface. A tooth immersed in the juice of a lemon (citric acid) would soon lose its polished surface and assume a chalk-like appearance, and if let alone long enough would undergo a partial decalcification. Common cider vinegar (acetic acid) will corrode the surface of the enamel in forty-eight hours. If you would note the action of acid upon carbonate of lime, place a little lemon-juice or strong vinegar upon a marble slab and allow it to remain twenty-four hours. The polish is first destroyed, and then white powder may be scraped from the spot after the acid has evaporated. Dr. Brophy has spoken of the alkalinity which is shown to exist so frequently in some mouths. This may be true of some portions of the mouth. For instance, the product of the sub-maxillary, sub-lingual and parotid glands may show, as they accumulate in the floor of the mouth, but if litmus paper were put into the cavities of carious teeth or in spaces where food has accumulated, the test would doubtless show an acid reaction.

Fermentation is the great factor in producing caries of the teeth, and cleanliness, absolute cleanliness, about the only preventive. Dr. Miller has found that the micro-organisms of caries do not flourish in carbolic acid or bichloride of mercury. Neither are they in their element when in the forms of tobacco or in a decoction of that weed.

Dr. J. Curtis would like to know if the microbes do the work of destruction themselves, or do they generate an acid which does. He believes we always change the shape of a tooth when we fill it, or at least that we change the conditions which surround it, and that is why, when the work is properly done, the decay does not recur. The surfaces are made smoother, so that they can be kept clean more readily, and thus prevent fermentation from taking place.

Dr. John Van Duyn, Syracuse, addressed the convention on the subject of the "Abnormity of the Dentist's Eye." He said:

"The eye is the most important instrument which the dentist has. In some callings the hearing or other faculties are more useful, but in the practice of dentistry the eye is the all-important. In proportion as the eye of the dentist is defective, his usefulness is lessened. Some persons have trouble with their eyes from birth, others have difficulties which are acquired at various periods of their lives, at thirty, forty or fifty years. He would not go into a list of the troubles of the eye, but would only state a few of them. These are connected with refraction,—with the way the rays of light are disposed of after they enter the eye. [Dr. Van Duyn here drew upon the blackboard a diagram of the normal eye, which he explained.] When a ray of light enters the normal eye, what becomes of it? Those rays which strike the centre go straight through; if they strike above or below the centre they are refracted—he would not speak of the laws of refraction—so that all meet at a point upon the retina, which is called the focus. Such an eye is called emmetropic. When the light rays are refracted in this way the eye sees, and vision is perfect. In an eye in which the antero-posterior portion is shortened, the focus comes behind the retina, and it is characterized by indistinct sight, the hypermetropic eye. There is another condition, just the opposite of this, in which the antero-posterior portion of the eye is too long. Here the light-rays focus before they reach the retina. This is the near-sighted, the myopic eye. There is still another condition, of which Dr. Marshall can tell you something, the irregular eye, the astigmatic eye. In this the meridian in one eye is normal, or hypermetropic, or myopic, and in the other, one of the other conditions is found. Any two of them may be combined. If it is a regular astigmatism—where one meridian is normal—the difficulty can be perfectly corrected by properly made glasses; but when it is irregular,—when neither eye is normal—the correction can be only approximate. In this eye the focus is not a point but a line.

The correction of all these difficulties of vision is of course by means of glasses. When the eye has its focus too far back, the lines of the rays of light must be made to converge before they enter the eye; so we put on a convex glass. For the myopic eye, the focus must be thrown back; hence concave glasses are used to cause the rays to diverge, so that when they enter the eye they will be carried further back than they would be without the glasses.

The hypermetropic and the astigmatic eye are congenital; the myopic is or it is not congenital. In the former case it is known; in the latter it may go on for years before the fact is discovered. When it is acquired or congenital the way to find it out is by comparison with normal sight. If it is found that the lens of the eye is imperfect so that one does not see, it must be changed. The sailor's eye, the keen vision of which is so often quoted, is not so good as the landsman's. The sailor will announce that the ship is approaching land long before the landsman aboard observes any sign of it. But it is not superior sight on the part of the sailor. He sees a mistiness, which his experience teaches him means that the land is near. With the hypermetropic eye there is not always a necessity for glasses, as when the condition exists in the young eye, because at five, ten, fifteen, or twenty years the lens is so soft in the lamellæ and the cillary muscles so strong that a greater curve is given to the lens when looking at near objects, so that the defect is not noticeable, although the act of seeing in such cases is accompanied by muscular effort. As the person grows older the lens becomes harder and is less easily curved, so that at the age of thirty to forty or forty-five years the muscular effort required begins to be felt as a strain and glasses are required. Every eye becomes hypermetropic at the age of seventy or eighty years; that is, it loses the power of curving the lens. This curving of the lens is what is called "accommodation." When we look at an object at a distance and then at one nearer; the lens is curved more for the nearer object.

Applying these general principles to the eye of the dentist, it is readily seen that a fissure in the enamel so fine as is often found will require strain of the eyes to see it; also, how important for the discovery upon the surface of the smallest points of the beginning of caries is perfect vision. Then, too, how is the approach of disease as disclosed by the shadows, by the depth of color, which we get by transmitted or reflected light, to be seen without it? All these things are only appreciated by the finest vision. Two luminous points cannot be distinguished by the normal eye except they are separated by an angle of sixty seconds; within that distance they seem as one. A few eyes can distinguish the difference at fifty seconds' separation, while others are so coarse that they require an interval of eighty or ninety seconds. In order to know, one must see, and persons who have not a sufficiently sensitive retina cannot know because they cannot see. Accuracy of vision depends upon the sensitiveness of the retina.

To go back to the emmetropic eye; let a man use the naked eye till he is forty years old in a pursuit which requires constant close application of sight, as in the practice of dentistry, and he will begin to feel the effects of fatigue on the eye. Sometimes the disturbance is in the eye itself, sometimes it is shown by headaches, and even by general weariness of the whole system. He is not sick, but he tires easily. This is due to the fact that the ciliary muscle is not able to do its work as formerly, and the strain is reflected. Just as soon as this occurs the retina loses sensitiveness, and just as soon as the sensitiveness is lost we do not see. So that it becomes necessary for a man intending to adopt the practice of dentistry to recognize that he must have perfect eyesight; and it follows as a corollary that no one should enter the profession without first having a competent examination of his eyes. If he has astigmatism of a kind which cannot be corrected by the use of glasses, he should refrain from entering the profession. Again, if, having become a dentist, he finds, after reaching the age of thirty years, that his eyes become fatigued, he should periodically repair to the proper authority for examination. By such a course only can he avoid the troubles which are to be attributed to over-use of the eyes.

Dr. John S. Marshall, Chicago: Prof. Van Duyn never said a truer word than when he declared that the eye was the best instrument of the dentist. The dentist is usually very sensitive about his eyes. How many of them put on glasses willingly? Dr. Marshall presumed that he was born with astigmatism. He was troubled with fatigue of the eye and general weariness all through his student life, and for twelve years after entering upon the practice of dentistry. He was completely broken down, and finally it was suggested that there might be trouble with his eyes. Prof. Van Duyn made an examination which disclosed the existence of horizontal stigmatism, and he prescribed the very glasses which the speaker has on to-day. There are probably many here to-day who suffer from these troubles of the eye. They cannot get near enough to the patient's mouth to see the work properly, and when the day's work is done they go to their homes feeling worn out. A gentleman near Chicago thinks that dentists should use different glasses from those usually prescribed. He thinks they should wear prismatic glasses, because, as he says, the dentist has to get so close to his work that the normal eye cannot see without injury, and the effort causes a slight convergent squint. If they would get prismatic glasses the eye would look straight out. Dr. Black wears them with much satisfaction. If dentists would lay their pride aside and wear glasses whenever they are needed, they would be better off and their patients very much so. He had tried to get along with using them only a part of the time. One day he would wear them and feel no trouble. The next day without them he would feel the old sense of fatigue. Since wearing them continuously he had had no trouble. It is probable that some persons go to the age of fifty years without losing the power of accommodation. The majority of dentists probably suffer from some form of eye-trouble. It is likely that very few who have been in practice for ten years have perfect eyes. The reason for this is that a good deal of the dentist's work is done in the back of the mouth, where the light is poor under the most favorable circumstances.

Dr. Van Duyn thought that this would hardly correct the evil. It is probable that many of the defects of vision from which dentists suffer existed long before they became dentists, which only makes true what he had said as to the importance of the dentist knowing exactly what his eyes are.

Dr. J. Branston Willmott, Toronto, Canada, would not venture to speak upon the subject, but that possibly the relation of his personal experience might prevent others from suffering a similar penalty for want of knowledge. He was born, he presumed, with a defect of vision,—congenital astigmatism,—but he did not find out until he was thirty-eight years of age. His eyes did not focus together, and when his sight began to fail he was troubled with a great twitching of the muscles of one eye, accompanied by considerable weakness. Before night came each day he would be wearied out, and sleep failed to restore him to his normal condition. His eyes were examined by an oculist, the difficulty discovered, and glasses were made to correct the astigmatism. The twitching ceased in one day after he began to wear them, and his health was soon restored to its normal tone. What was singular about the case was that about two years afterward the prescription glasses were broken, and since then he had used ordinary, though strong glasses, without any recurrence of the former symptoms. The wearing of the special glasses seemed to have cured his trouble entirely. He related these facts merely to emphasize the wisdom of consulting a specialist on the first indication of failing or defective vision.

Dr. Truman W. Brophy, Chicago, felt more than usual interest in the subject under discussion, because of the experience he had passed through during the past three or four years. Some four years ago his eyes began to grow weary toward the latter part of the day, and especially along towards the end of the week. He procured glasses, but the examination was not critical, and later it had to be done over. With the first glasses he found that on Monday he did not need them; on Tuesday he used them a part of the day, and on Wednesdays and Thursdays from the time he began operating in the morning. During the remainder of the week he was troubled with headaches as before, in spite of the glasses. After two months of this kind of experience, he underwent a critical examination by a well-known oculist, who pronounced his eyes hypermetropic, and prescribed the proper glasses. The headaches disappeared at once on beginning to wear them, and he felt far better than for years. It would seem to be the part of wisdom for those who use the eyes much to get glasses early, not only to avoid distressing physical symptoms, but, what is of more importance, to preserve their eyesight through life. Prof. Van Duyn says that hypermetropia is usually congenital. The speaker is satisfied that he had none of it in his earlier years. One thing to which he would direct special attention as of the highest importance to dentists,—the necessity of not using the eyes in a bad light, as towards night. In having glasses made do not have the bows too tight, as by pinching the nose they will cause headache, but have them made to fit on loosely.

Dr. W. H. Dwinelle, New York: As to the question whether a large magnifying glass would correct the difficulty of poor sight, it might do so for the normal eye which was simply growing old; but it will do no good for the astigmatic eye. This trouble is only to be corrected by glasses for the eye. It has been stated that as we grow old the focus of the eye is carried farther back. It sometimes occurs that the sight of old persons is partly restored to the normal condition. This he had observed among several of his patients, one of whom was in his office recently, and her eyes were tested in reading the finest type used in printing. The theory is that in such cases the retina to some extent takes up the office of the ciliary muscles and so partially restores the lens to its normal condition. We can thus take up the office of the ciliary muscles at any time to some extent, and on this principle some have recommended the manipulation of the eye as a hygienic measure. John Quincy Adams always manipulated his eyes, and he never wore glasses. Dr. Dwinelle is satisfied from experience with his own eyes that this theory has at least a foundation in fact. We know that in the old the eye becomes flattened which throws the focus out of place, and we can almost recognize near-sighted persons by the shape of the eye, which is much more rounded in the outward contour than the normal eye.

Dr. Marshall wished to offer a suggestion which followed out will afford considerable rest to the eyes during an operation. We all know that when the eye is kept fixed on any one point for a considerable length of time, and more especially if the gaze is intent, it is very fatiguing to the muscles. If the dentist's office is so arranged that he can once in a while look away from his work to some object at a distance, he will find that from this simple act his eyes will be rested; and if he can make this a habit he will be very much benefited.

Dr. Brophy wanted to ask Prof. Van Duyn a question, and he would preface it by the statement that he thought the cause of the trouble with his eyes was a cross-light in his office. The question he would ask is, What light is best for the dentist, and is a cross-light detrimental ?

Dr. Van Duyn. The best light comes not direct from the sun, but from a luminous sky or a light cloud. A north light from a cloud is best of all. A cross-light should never be used.—*Cosmos.*

